# The dual guardians of cellular stability: exploring nesprin and lamin in senescence

**DOI:** 10.1038/s41419-025-08087-w

**Published:** 2025-10-24

**Authors:** Zi-yi Zhou, Qin Qin, Cengyuan Dong, Yangyuanzhi Liu, Chunyu Cao, Lin Teng

**Affiliations:** 1 The First Clinical Medical College,Three Gorges University/Department of Cardiology, Yichang Central People’s Hospital, Yichang, 443003 Hubei PR China; 2 School of Basic Medicine, China Three Gorges University, Yichang, 443000 Hubei PR China; 3 College of Basic Medical Sciences, Hubei Key Laboratory of Tumor Microencironment and immunotherapy, China Three Gorges University, Yichang, 443000 Hubei PR China; 4 Institute of Cardiovascular Diseases, Three Gorges University, Yichang, 443003 Hubei PR China

**Keywords:** Senescence, Dynein

## Abstract

Cellular senescence is a state where cells permanently exit the cell cycle after a finite number of divisions, while maintaining metabolic activity. This phenomenon, initially described by Leonard Hayflick, plays a pivotal role in aging, contributing to the progressive decline in physiological function by promoting tissue dysfunction and restricting regenerative capacity. It is regulated by an array of factors, including DNA damage, telomere shortening, oxidative stress, mitochondrial dysfunction, and epigenetic modifications. Nesprins, a family of transmembrane proteins embedded in the nuclear envelope, are integral components of the LINC (Linker of Nucleoskeleton and Cytoskeleton) complex, which connects the nucleus to the cytoskeleton, thus preserving structural integrity and facilitating mechanotransduction. Lamin proteins, which form the nuclear lamina beneath the inner nuclear membrane, provide support to nuclear envelope architecture, organize chromatin, and modulate gene expression. Lamin proteins also interact with nesprins to collectively sustain nuclear mechanics and maintain morphological stability. Understanding the molecular mechanisms by which nesprins and lamins influence cellular senescence provides valuable insights into the biology of aging and may offer novel therapeutic avenues to address age-related diseases. This review examines the interactions between nesprin and lamin proteins and their potential contributions to cellular senescence.

## FACTS

- Nuclear membrane proteins, such as lamins and nesprins, are crucial for maintaining nuclear structure and function, and their dysfunction is linked to aging.
- The LINC complex, formed by nesprins and SUN proteins, connects the nucleus to the cytoskeleton, playing a role in mechanotransduction and nuclear positioning.
- Mutations in lamin A/C are associated with progeria syndromes, which are models for studying aging.
- Nesprins are involved in DNA damage response, mechanotransduction, and mitochondrial function, all of which are related to cellular senescence.
- The interaction between nesprins and lamins through the LINC complex is essential for nuclear stability, force transmission, and DNA repair.

## OPEN QUESTIONS

- How do specific mutations in lamin A/C and nesprins contribute to the molecular mechanisms of aging and age-related diseases?
- What are the precise mechanisms by which the LINC complex influences DNA damage response and repair in aging cells?
- Can targeting nuclear envelope proteins like lamins and nesprins be a viable strategy for developing therapies against age-related diseases?

## Introduction

**Fig. 1:**
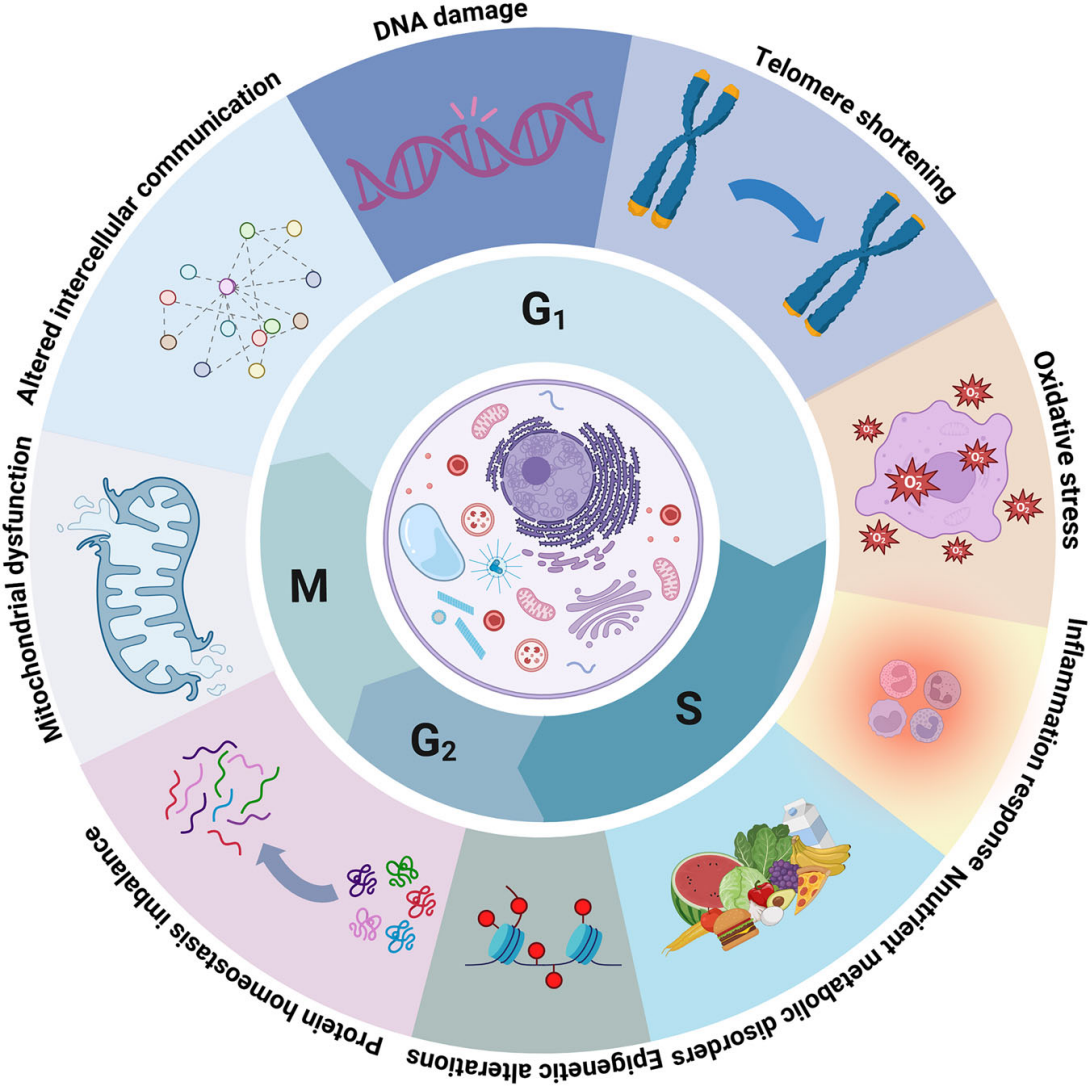
Key changes in the cell life cycle and their influencing factors. Multiple biological challenges and markers of senescence that cells may face at different stages (G1, S, G2 and M phases) are illustrated. These factors include: DNA damage, telomere shortening, oxidative stress, inflammatory responses, nutrient metabolism disorders, epigenetic alterations, protein homeostasis imbalance, mitochondrial dysfunction, and altered intercellular communication. These factors interact with each other and together affect cell function and longevity, potentially leading to an acceleration of the aging process. Created with BioRender.com.

Recent advancements in aging research have revealed that aging is not simply a chronological process but a dynamic cellular state driven by various physiological mechanisms contributing to multiple age-related diseases. Aging results from a complex interplay of intrinsic and extrinsic factors, rather than a single cause [1, 2]. Cells can only divide a limited number of times in vitro and irreversibly stop dividing, a phenomenon now considered the *Hayflick limit* [3–7]. Aging is a multifaceted process that leads to a progressive decline in the function of organs and tissues across the body [8–10]. To define aging, three critical criteria have been established [1]: traits must correlate with aging [2]; experimental induction of these traits should accelerate aging; and [3] interventions targeting these traits should be able to slow, halt, or even reverse aging [11]. Current research has identified nine key factors that contribute to aging: DNA damage, telomere shortening, oxidative stress, inflammatory responses, nutrient metabolism disturbances, epigenetic changes, protein homeostasis imbalance, mitochondrial dysfunction, and altered intercellular communication (Fig. 1) [4, 11, 12].

**Fig. 2:**
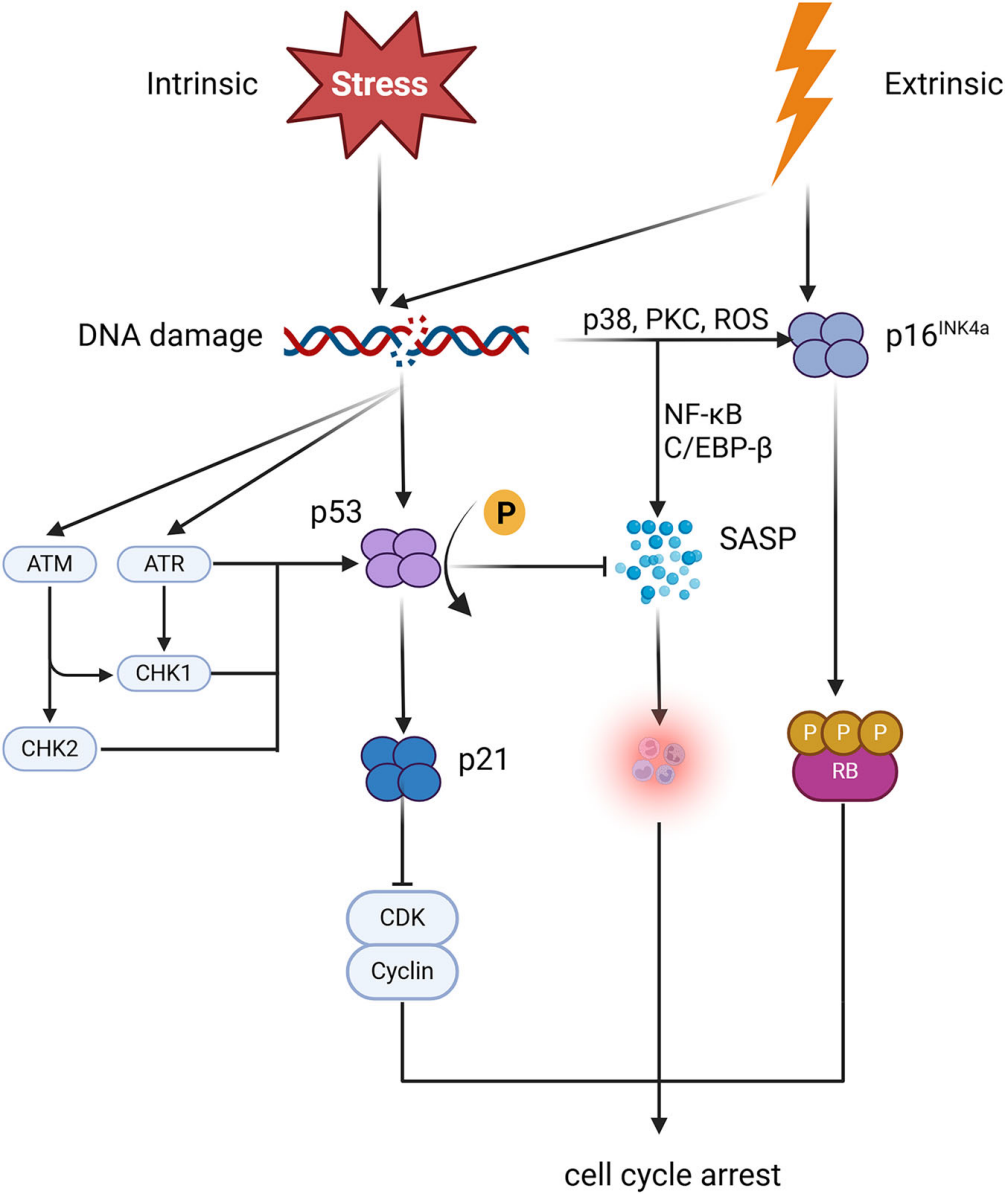
Diagram of cellular senescence pathways in response to intrinsic and extrinsic stress. The diagram illustrates cellular responses to both intrinsic and extrinsic stressors leading to cell cycle arrest and senescence. DNA damage activates the ATM and ATR kinases, which in turn activate CHK1 and CHK2 kinases, leading to the stabilization and activation of p53. Activated p53 induces the expression of p21, a CDKI that blocks the CDK-cyclin complex, resulting in cell cycle arrest. Extrinsic factors such as ROS and stress-activated protein kinases (e.g., p38, PKC) also contribute to the activation of p16^INK4a^. The activation of p16^INK4a^ results in the inhibition of RB, which promotes cell cycle arrest. Additionally, these pathways can lead to the secretion of pro-inflammatory factors through the SASP (senescence-associated secretory phenotype), mediated by transcription factors like NF-κB and C/EBP-β, further reinforcing the senescent state. Created with BioRender.com.

One of the most critical cellular senescence triggers is the DNA damage response (DDR) activation. This complex system that detects and responds to molecular damage through genetic and stress signaling pathways [13–15]. DDR activation, often initiated by telomere shortening or other forms of cellular dysfunction, frequently leads to senescence. When the extent of damage exceeds the cell’s capacity for repair, the cell enters a stable, irreversible state of cell cycle arrest [16]. The triggers of cellular senescence can be broadly categorized into intrinsic and extrinsic factors. Intrinsic factors include DNA damage, genomic instability, telomere shortening or dysfunction, oncogene activation, epigenetic alterations (such as histone modifications that activate the p16-Rb signaling pathway), cell fusion, and oxidative stress mediated by reactive oxygen species (ROS). Extrinsic factors encompass environmental influences, including exposure to ultraviolet (UV) and ionizing radiation [17–19]. These stressors activate multiple molecular pathways, contributing to the onset of senescence [20]. In response to these stress signals, p53 is activated through several mechanisms, including the phosphorylation and stabilization of kinases, leading to the expression of cyclin-dependent kinase inhibitors (CDKIs), such as p21^CIP1^ [21, 22]. Elevated p21^CIP1^ levels inhibit cyclin-dependent kinases (CDKs), resulting in permanent cell cycle arrest. Additionally, in response to cellular damage and stress, the normally low expression levels of p16^INK4a^ in healthy tissues can become dysregulated. p16^INK4a^ functions as a tumor suppressor by halting cell cycle progression and promoting senescence. Specifically, p16^INK4a^ inhibits the activity of CDK4 and CDK6, proteins involved in the phosphorylation and inactivation of the Rb protein. This disruption of Rb regulation leads to irreversible cell cycle arrest (Fig. 2).

While losing the gene expression necessary for proliferation, senescent cells acquire distinct characteristics and functions that distinguish them from non-senescent cells. These changes include irreversible growth arrest, the formation of DNA damage foci, and alterations in the epigenetic profile, such as the development of senescence-associated heterochromatin foci (SAHFs). Further changes in nuclear membrane composition are observed, including reduced levels of lamin B1 and elevated levels of high-mobility group box 1 (HMGB1). Senescent cells also exhibit increased levels of CDKIs like p21^CIP1^ and p16^INK4a^, alongside the accumulation of senescence-associated lipids, such as ceramides. Other hallmark features of senescence include remodeling of the cell surface, mitochondrial dysfunction, increased ROS production, disruption of signaling pathways, impaired protein homeostasis, resistance to apoptosis, and defective autophagy. Elevated activity of lysosome-related senescence-associated β-galactosidase (SA-β-Gal) suggests increased lysosomal mass and activity [23, 24]. Moreover, senescent cells often exhibit the senescence-associated secretory phenotype (SASP), characterized by the release of a variety of molecules, including pro-inflammatory cytokines, chemokines, growth factors, proteases, damage-associated molecular patterns (DAMPs), and extracellular matrix components, such as proteins and exosomes [17, 25–29]. The composition of SASP factors varies significantly depending on the inducing stimuli, the cell type undergoing senescence, and the surrounding microenvironment (Table 1) [24, 30].

**Table 1:** Some of the main factors that make up SASP.

Factors	Relationship with cellular senescence	Major regulatory pathways	Function and impact
IL-1α	Acts as an early pro-inflammatory factor and is a key component of SASP, amplifying and maintaining cellular senescence	NF-κB, p38 MAPK,mTOR	Induction of secretion of other inflammatory factors and SASP components amplifies senescence signals, activates immune cells, accelerates peripheral cells into a senescent state, and increases the chronic inflammatory burden on tissues
IL-1β	Highly expressed in senescent cells, triggers and amplifies the SASP effect, which is important for increasing inflammation in the surrounding environment		Increases the incidence of chronic inflammation, attracts immune cells to clear senescent cells, degrades the ECM, induces tissue remodeling, and accelerates tumor progression in certain tumor settings
IL-6	Promotes cells to enter an irreversible state of senescence and is one of the key factors in the chronic inflammatory response	JAK/STAT, NF-κB,mTOR	By inducing cell cycle inhibitory proteins to accelerate the senescence process, enhancing the secretion of other SASP molecules, activating the immune response, creating an ‘inflammatory senescence’ environment, and interacting with the cancer cell microenvironment
IL-8	Acts as an important chemokine, attracting immune cells to reach the periphery of senescent cells, while promoting its own senescence through an autocrine mechanism	NF-κB, p38 MAPK,mTOR	Attracting immune cells such as neutrophils to remove senescent cells maintains a chronic inflammatory environment, alters intercellular communication, increases the likelihood that surrounding cells will enter a senescent state, and exacerbates tumor growth and metastasis
IL-10	Anti-inflammatory cytokines, although anti-inflammatory, inhibit excessive inflammatory responses through immunomodulation in the periplasmic environment of senescent cells	JAK/STAT	Suppresses excessive inflammatory responses and balances the immune system’s overreaction to senescent cells, but may lead to immune escape in the tumor microenvironment, promoting tumor survival and expansion
TNF-α	Pro-inflammatory factors that activate other SASP components in senescent cells and participate in the feedback loop of the inflammatory response	NF-κB, JAK/STAT	Increased secretion of the SASP molecular population regulates cell survival and apoptosis, and prolonged action induces chronic inflammation and promotes the development of the microenvironment for degenerative diseases and cancer
TGF-β	Regulates cell cycle and growth inhibition, participates in tissue repair but can lead to tissue fibrosis when secreted continuously	SMAD, p38 MAPK	Promotes cell cycle arrest, fibrosis and ECM remodeling, and contributes to repair after tissue injury, but may exacerbate fibrotic lesions and accelerate ageing in chronic inflammation
MMPs	Alteration of the tissue microenvironment through degradation of ECM structures and extracellular matrix, with profound effects on tissue architecture	NF-κB, AP-1	Degradation of the extracellular matrix, disruption of normal tissue structure and enhancement of cell migration contribute to the infiltration and metastasis of tumor cells and further diffuse the SASP effect during the inflammatory process
VEGF	Promote neovascularisation and enhance tissue access to nutrients, especially in the tumor environment Increase vascular density	PI3K/AKT, p38 MAPK	Supports neovascularisation, provides nutrient supply for tumor growth, promotes cell proliferation and migration, and in healthy tissues may lead to abnormal angiogenesis and tissue structural abnormalities
GM-CSF	Stimulates immune cell activation, increases the persistence of the inflammatory response and helps the immune system to recognize senescent cells	JAK/STAT, NF-κB	Activates macrophages and neutrophils, enhances immune system clearance and helps to remove senescent cells, but prolonged secretion can lead to chronic inflammation and accelerate tissue damage
CXCL1	Chemokines that attract immune cells such as neutrophils to the site of senescent cells and support the local inflammatory environment	NF-κB, p38 MAPK	Attracting immune cells to migrate to the site of senescent cells maintains the activity of the inflammatory milieu, helping to clear senescent cells, and in the case of tumor cells may create a protective microenvironment
CCL2	Chemotaxis of monocytes and macrophages to the periphery of senescent cells to further amplify the chronic inflammatory response	NF-κB, JAK/STAT	Supports chronic inflammation, enhances immune cell clearance, can promote immune escape in the cancer microenvironment and supports tumor progression through microenvironmental regulation
CCL5	Attract macrophages and lymphocytes to the vicinity of senescent cells and participate in the immune response and maintenance of chronic inflammation	NF-κB, JAK/STAT	Enhances migration of immune cells and helps to remove senescent cells, but may also lead to chronic inflammation and tissue damage, which in the tumor environment may provide an aid to tumor growth
IGFBP	Binds IGF, regulates IGF signaling and influences cell proliferation and metabolism	IGF-1/AKT	Regulates the activity of growth factors, affects cell proliferation and metabolism, participates in the regulation of the metabolic environment in aging and tumors, and promotes cell proliferation and growth in tumors
ROS	Enhances DNA damage and oxidative stress, inducing cellular senescence and creating an ‘oxidative senescence’ environment	NADPH oxidase, mitochondrial	Inducing DDR through increased oxidative stress triggers cell cycle arrest and senescence, driving the degenerative processes of cells and tissues and contributing to the development of chronic diseases
PGE2	Involvement in inflammation and angiogenesis via the prostaglandin pathway and influence on extracellular matrix remodeling	COX-2, EP receptor	Promotes local inflammatory responses, enhances angiogenesis, supports tumor growth and expansion, affects tissue structure by modulating ECM remodeling, and causes chronic inflammation with long-term secretion
GRO-α	Chemokines that attract neutrophils to the periphery of senescent cells and maintain the local inflammatory environment	NF-κB, p38 MAPK	Attracting immune cells involved in senescent cell clearance and maintaining a chronic inflammatory environment may have a supportive effect on tumors, increasing metastasis and expansion
Angiotensin II	Regulates vasoconstriction, increases blood pressure, and exacerbates aging-related diseases such as atherosclerosis in a chronic inflammatory environment	RAAS, NADPH oxidase	Increases vascular tone, contributes to the expansion of chronic inflammation, increases the risk of diseases of the cardiovascular system and accelerates the process of atherosclerosis, which has an aggravating effect on high blood pressure and diseases associated with aging
Exosomes	Regulates intercellular communication by interacting with target cells	NF-κB,p53/p21,mTOR,cGAS-STING	Transmit senescence signals, drive chronic inflammation, and remodel the tissue microenvironment through ECM degradation and fibrosis,also induce DNA damage in neighboring cells, accelerate senescence, and promote tumor progression through angiogenesis, immune evasion and metastasis

*ECM* extracellular matrix, *NF-κB* nuclear factor-kappa B, *MAPK* mitogen-activated protein kinase, *JAK/STAT* janus kinase/signal transducer and activator of transcription, *PI3K/AKT* phosphoinositide 3-pinase/protein kinase B, *ROS* reactive oxygen species, *RAAS* renin-angiotensin-aldosterone system, *NADPH* nicotinamide adenine dinucleotide phosphate, *COX-2* cyclooxygenase-2, *EP receptor* eicosanoid receptor, *AP-1* activator protein 1, *IGF-1* insulin-like growth factor 1, *TGF-β* transforming growth factor-β, *IL* interleukin, *TNF-α* tumor necrosis factor-α, *MMP* matrix metalloproteinase, *VEGF* vascular endothelial growth factor, *GM-CSF* granulocyte-macrophage colony-stimulating factor, *CXCL* chemokine (C-X-C motif) ligand, *CCL* chemokine (C-C motif) ligand, *IGFBP* insulin-like growth factor binding protein, *PGE2* prostaglandin E2, *GRO-α* growth-regulated oncogene-α.

Nuclear membrane proteins are essential for maintaining nuclear architecture, regulating gene expression, and facilitating signal transduction. As aging progresses, the expression levels and functional integrity of these proteins are significantly altered, resulting in nuclear envelope instability [31–35]. This instability is linked to abnormal nuclear morphology, disruption of chromatin organization, and impaired DNA repair capacity [36–39]. Changes in nuclear membrane proteins can also exacerbate cellular stress responses, further accelerating the functional decline associated with aging [31, 37, 40]. Given their pivotal role in nuclear structure and genome regulation, it is crucial to understand how specific nuclear envelope proteins contribute to senescence [4]. This review will focus on selected nuclear membrane proteins, such as nesprins, to elucidate their mechanistic involvement in cellular aging and explore their potential as therapeutic targets [36, 41–43].

## Lamin

**Fig. 3:**
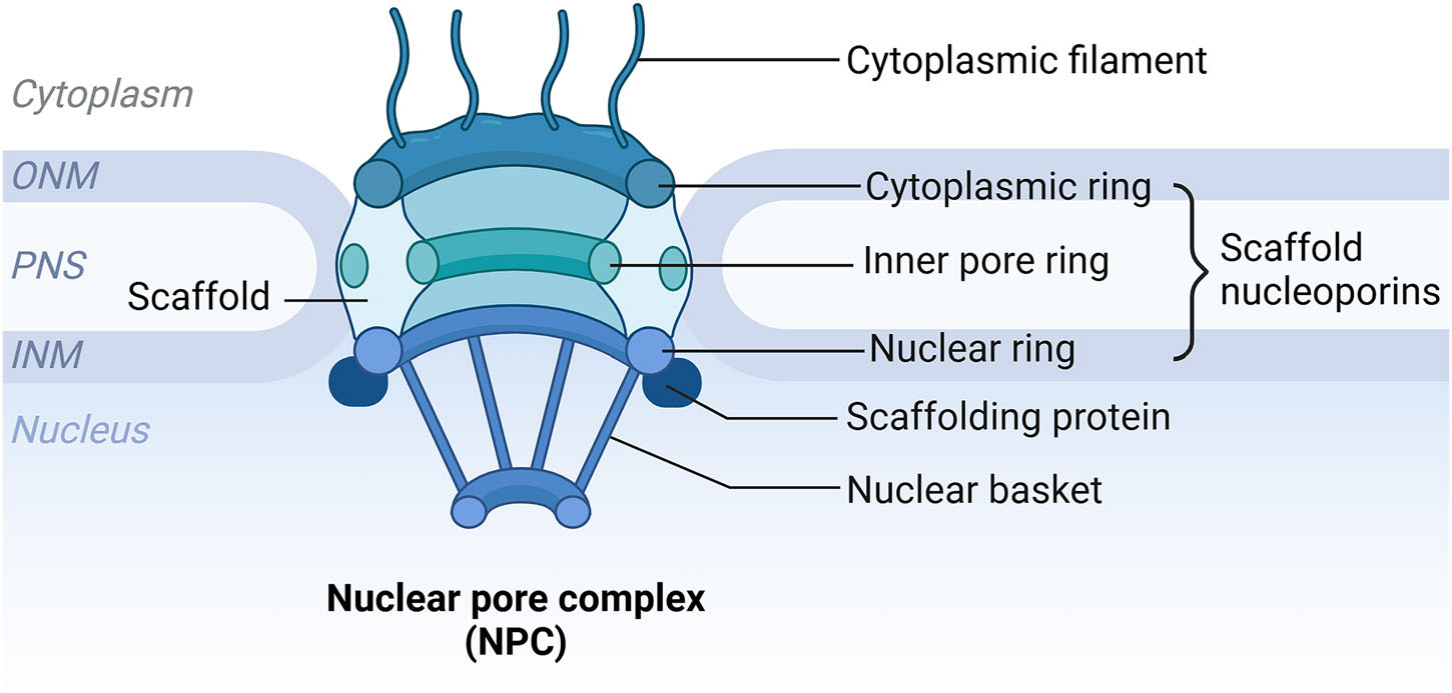
The structure of the NPC. This diagram illustrates the NPC, a crucial cellular structure facilitating molecular transport between the nucleus and cytoplasm. Key components include: Cytoplasmic filament: extends into the cytoplasm, aiding in transport processes; Cytoplasmic ring: part of the scaffold nucleoporins, located on the cytoplasmic side; Inner pore ring: located within the central part of the complex, also part of the scaffold nucleoporins; Nuclear ring: positioned on the nuclear side, connecting to the nuclear basket; Scaffold nucleoporins: structural proteins forming the core scaffold of the NPC; Scaffolding protein: provides additional support and stability to the structure; Nuclear basket: extends into the nucleus, involved in the selective transport of molecules. The nuclear pore complex spans both the ONM and the INM, traversing the perinuclear space (PNS) to connect the cytoplasm and the nucleus. Created with BioRender.com.

**Fig. 4:**
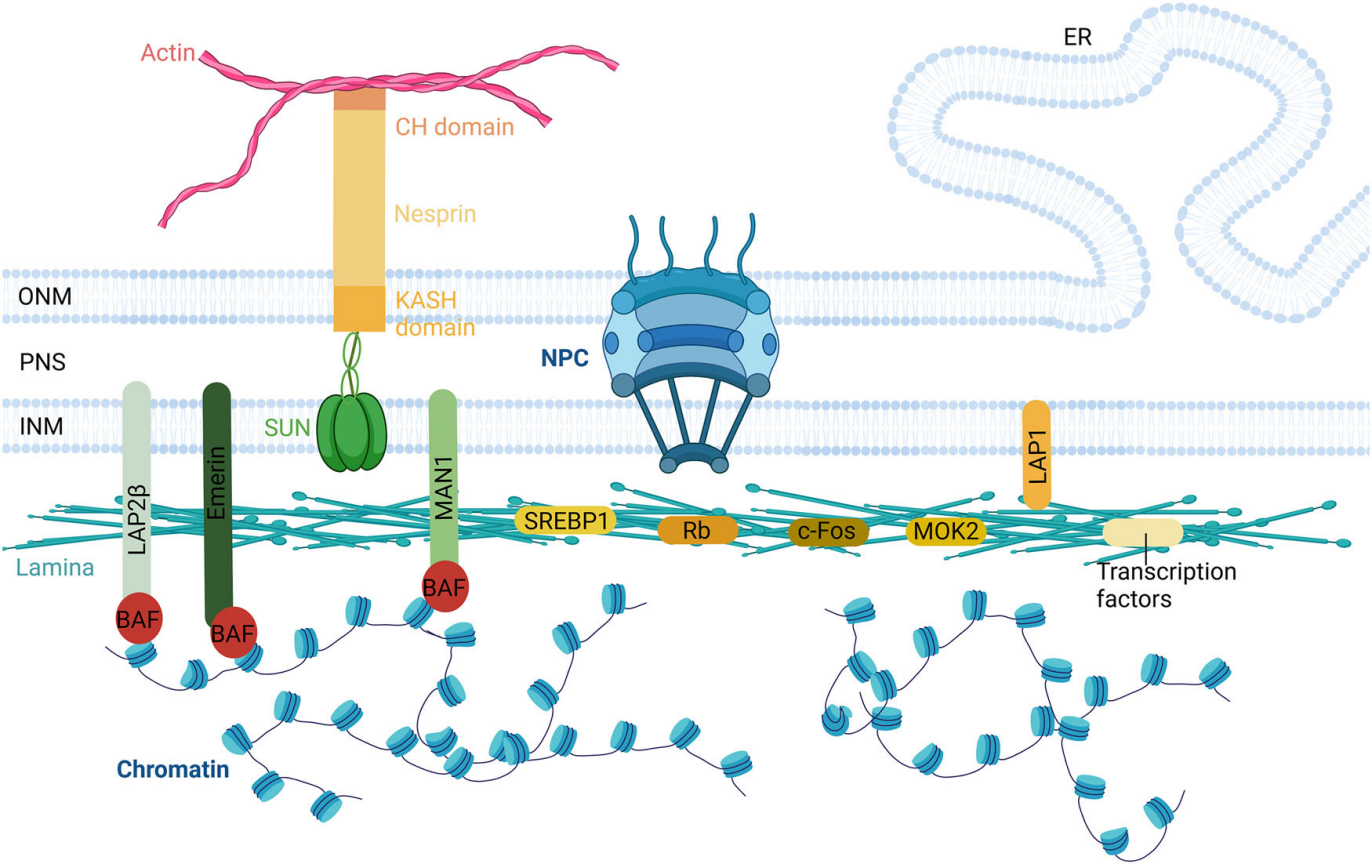
The structure and complexity at the nuclear envelope. The lamina is located under the inner nuclear membrane and consists of A-type lamin and B-type lamin. The lamina is used to maintain nuclear integrity and serves as a structural scaffold that anchors a variety of proteins and heterochromatin structural domains to the nuclear membrane (NE). Laminin-associated proteins (LAPs) and a number of nuclear membrane transmembrane proteins including LINC complex (nesprins containing the SUN and KASH structural domains), and LEM structural domain proteins (LAPβ, emerin, MAN1) interact with chromatin. The proteins that interact with lamin at the nuclear envelope are primarily believed to have mechanical and structural roles. Those that directly bridge lamin and chromatin are crucial for reinforcing the nucleoskeleton and for mechanically regulating gene transcription, while others are involved in regulating cell signaling. Created with BioRender.com.

The nucleus is the cell’s central organelle, serving as the repository of genetic material and regulating subcellular organization [44, 45]. The cytoskeleton mediates its dynamic interactions with various organelles, establishing critical connections essential for spatial organization [46–49]. In epithelial cells, the nucleus is typically positioned near the basal membrane through microtubule-driven mechanisms, whereas fibroblasts rely on actin filaments for nuclear positioning [50–52]. The nuclear envelope is a double-layered membrane structure surrounding the nucleus, consisting of the inner nuclear membrane (INM), outer nuclear membrane (ONM), and nuclear pore complex (NPC) [40, 53]. The inner and outer nuclear membranes are connected to the nuclear lamina and the endoplasmic reticulum, respectively, while the NPC regulates the exchange of materials between the nucleus and cytoplasm(Fig. 3) [54]. The nuclear envelope plays a crucial role in protecting genetic material, maintaining nuclear stability, regulating gene expression, and facilitating communication between the nucleus and cytoplasm(Fig. 4)[40, 53, 55].

**Fig. 5:**
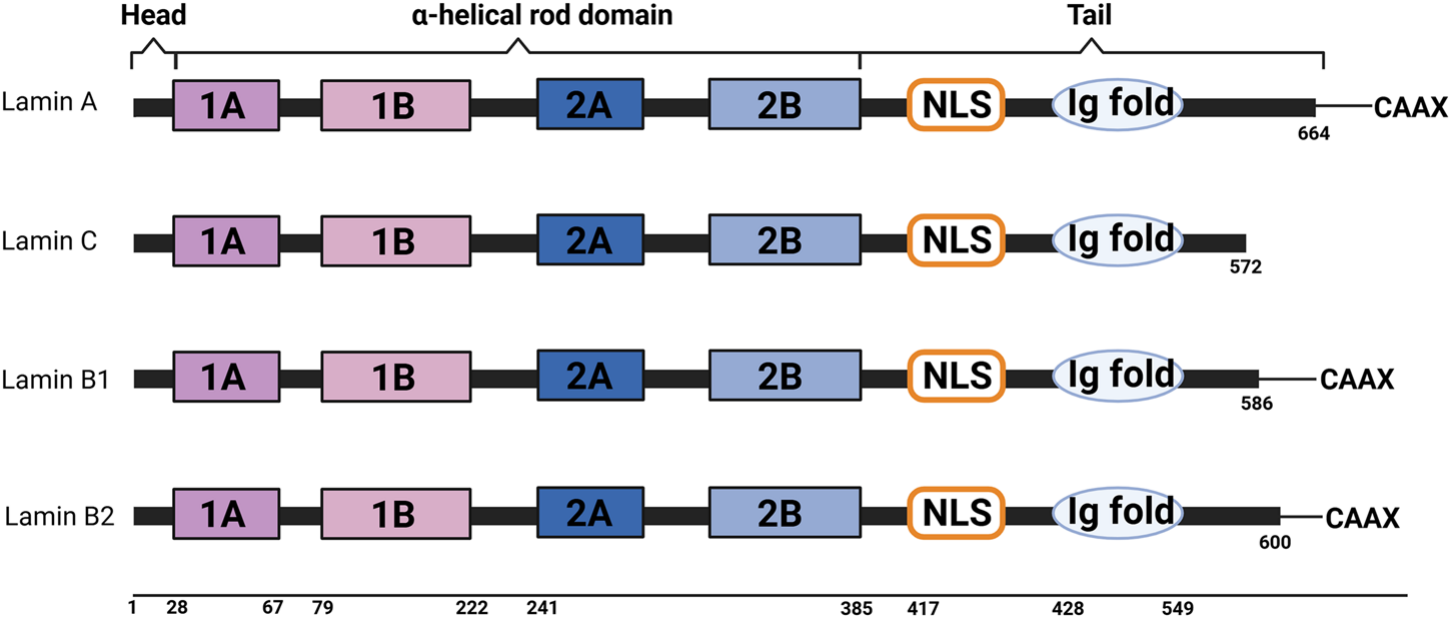
The structure of lamins. This diagram presents the domain organization of lamin family proteins, including lamin A, lamin C, lamin B1, and lamin B2. α-helical rod domain,comprising segments 1A, 1B, 2A, and 2B, this region facilitates dimerization and filament formation. Nuclear Localization Signal(NLS) enables the protein to be transported into the nucleus. Immunoglobulin-like fold(Ig fold) is important for structural stability. Tail contains the CAAX motif in lamin A, B1, and B2, which undergoes post-translational modifications for membrane association. Lamin C lacks the CAAX motif present in the other lamin proteins, affecting its localization and function within the cell. Created with BioRender.com.

Electron microscopy has revealed a fibrous protein mesh structure beneath the INM, which has been identified as lamin [56–58]. Recent research indicates that lamin belongs to the type V intermediate filament (IF) family [39, 59, 60]. Like other IF proteins, lamin consists of a central α-helical rod domain flanked by globular N-terminal “head” and C-terminal “tail” domains, the latter containing lamin-specific motifs, including a nuclear localization signal [61–63]. The central rod domain promotes the formation of coiled-coil dimers, which further assemble into higher-order structures essential for maintaining nuclear integrity and organization. Mammals possess three lamin genes, *LMNA*, *LMNB1*, and *LMNB2*, which encode four major and three minor lamin isoforms (Fig. 5) [64, 65]. The *LMNA* gene encodes A-type lamins, including the major isoforms A and C (with molecular weights of 70 and 65 kDa, respectively), and the minor isoforms A∆10 and C2. B-type lamins include the major isoforms B1, encoded by *LMNB1*, and B2, encoded by *LMNB2* (with molecular weights of 67 and 68 kDa, respectively), as well as the minor isoform B3 [66]. Lamin B1 and B2 are expressed in most cells of both embryos and adults. Their expression is critical for nuclear integrity, cell survival, and normal development [67–69]. In contrast, A-type lamins exhibit differential expression patterns and are typically associated with cell differentiation [67, 68, 70, 71]. In mice, A-type lamins are essential for development; however, *LMNA−/−* mice do not survive beyond 8 weeks of gestation [72]. Similarly, individuals lacking functional A-type lamins either die in utero or shortly after birth [73–75]. In contrast, cultured cells without lamin A/C can still divide normally [76]. The distinct expression patterns of A-type and B-type lamins suggest that B-type lamins are fundamental components of the nuclear lamina, whereas A-type lamins serve more specialized functions [77].

B-type nuclear lamins are essential components of the nuclear scaffold, a complex structure that provides the framework for nuclear organization and function. All B-type lamins are synthesized as precursors and undergo post-translational modifications to produce mature proteins. B-type lamins participate in a wide range of nuclear processes, including DNA replication and repair, chromatin regulation, and nuclear rigidity. Additionally, lamin B1 and B2 regulate cellular processes such as tissue development, cell cycle progression, cell proliferation, aging, and the DNA damage response. An increasing number of human diseases are linked to defects in lamin B1 or B2, contributing to the disorders known as laminopathies [42, 78]. Both A-type and B-type lamins have transcriptional regulatory functions. In embryonic and somatic cells, B-type lamins can bind to RNA polymerase II, and dominant-negative mutants can disrupt this interaction, inhibiting RNA polymerase II activity [79–82]. This suggests that B-type lamins play a role in RNA synthesis. In contrast, A-type lamins appear to influence the activity of transcriptional regulators. A-type lamins have been shown to interact with several transcriptional regulators, including MOK2 [83], sterol regulatory element-binding protein (SREBP1) [84], retinoblastoma protein (Rb), and c-Fos [85]. While the impact of A-type lamins on MOK2 and SREBP1 remains fully elucidated, their role in regulating Rb function is well established [85].

**Fig. 6:**
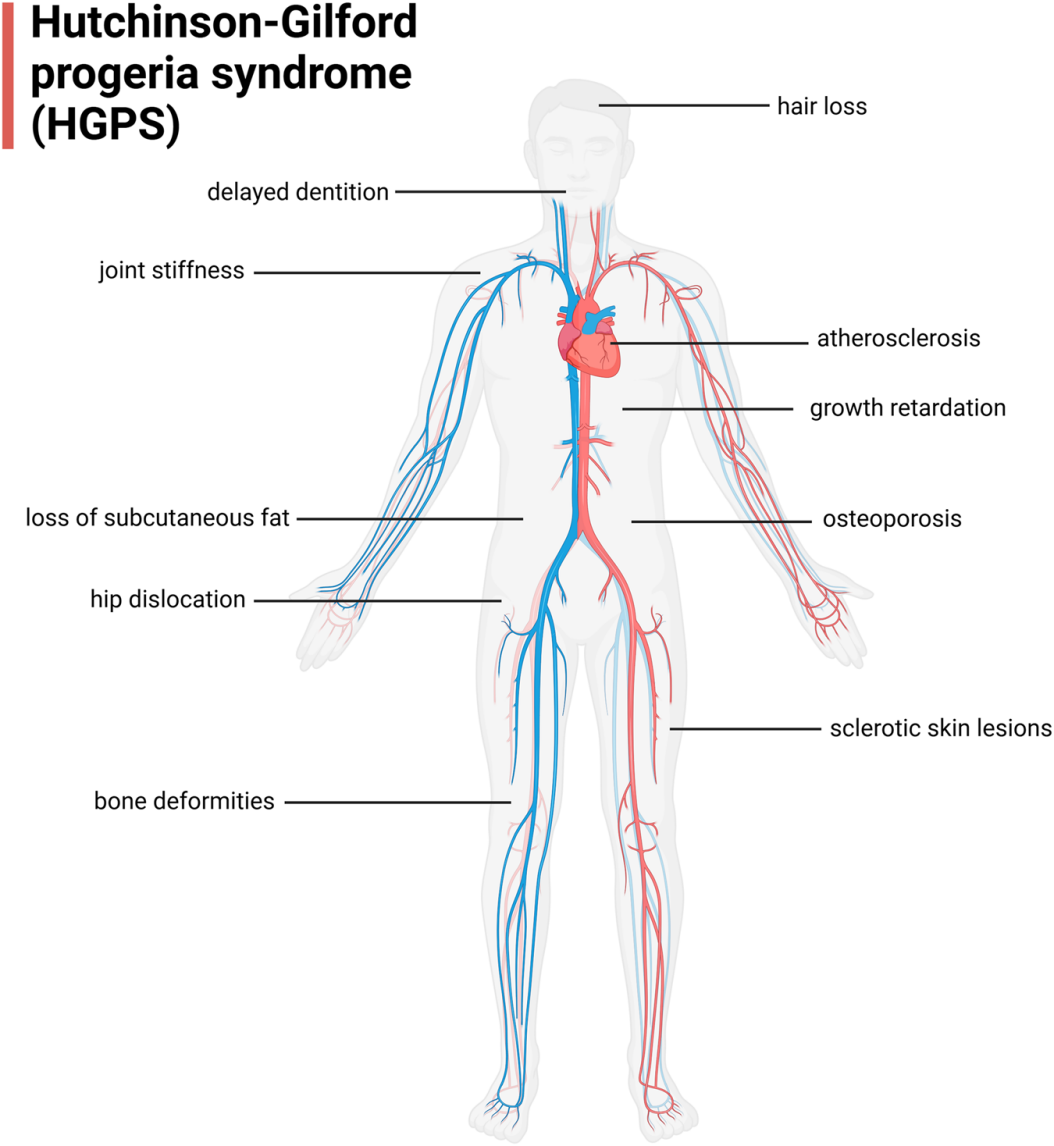
Clinical manifestations of HGPS. The image illustrates the common symptoms and physical characteristics associated with HGPS, including hair loss, delayed dentition, joint stiffness, growth retardation, atherosclerosis, osteoporosis, sclerotic skin lesions, loss of subcutaneous fat, hip dislocation, and bone deformities. Created with BioRender.com.

Hutchinson-Gilford progeria syndrome (HGPS) is a rare genetic disorder characterized by accelerated aging [86]. It serves as a model for studying the mechanisms of normal aging [87]. Children with HGPS typically appear normal at birth. However, within the first year, their condition deteriorates rapidly, manifesting in symptoms such as loss of subcutaneous fat, severe growth retardation, hair loss, bone deformities, osteoporosis, delayed dentition, joint stiffness, hip dislocation, sclerotic skin lesions, and progressive atherosclerosis(Fig. 6)[88, 89]. Patients with HGPS exhibit signs of premature aging, and in the disease’s final stages, most children develop hypertension, angina, and heart enlargement due to atherosclerotic heart disease. HGPS patients usually die around the age of 13 from myocardial infarction or cerebrovascular accidents [90]. The pathological basis of HGPS is a point mutation in the *LMNA* gene, which leads to the production of an abnormal form of lamin A known as progerin [91]. The abnormal production and accumulation of progerin are central to the pathogenic mechanism of this disease [92]. In HGPS patients, the *LMNA* gene typically undergoes a specific mutation, usually a C → T point mutation in exon 11, resulting in an abnormal splice site. This mutation leads to progerin production during the processing of prelamin A [93]. Normally, prelamin A undergoes farnesylation, methylation, and proteolytic cleavage to remove 15 amino acids from its C-terminus, forming mature lamin A [94]. However, in HGPS, progerin lacks this critical cleavage step, resulting in the retention of its farnesylated C-terminus, which prevents its proper incorporation into the nuclear membrane [95]. The accumulation of progerin leads to widespread damage to the cell nucleus [41]. Firstly, it disrupts the stability of the nuclear lamina, causing significant alterations in the nuclear structure [96]. The cell nucleus exhibits abnormal shapes, such as irregular contours and nuclear envelope invaginations, direct consequences of progerin expression [97]. Progerin expression leads to nuclear morphological abnormalities, chromatin reorganization, and genomic instability [98]. Research has shown that the accumulation of progerin significantly increases DNA damage, particularly in telomeric regions, pushing cells into an irreversible state of senescence [99]. This process closely mirrors the behavior of normal aging cells, suggesting that progerin expression is not only associated with HGPS but may also contribute to normal aging [100]. Another critical effect of progerin is its interference with interactions between nuclear envelope proteins and chromatin, which disrupts genomic stability [101]. Its abnormal interaction with DDR pathways leads to the accumulation of DNA damage, triggering cell cycle arrest and cellular senescence [102]. The normal function of lamin A in the nuclear lamina is replaced by progerin, preventing the cell from responding appropriately to both internal and external stress signals [101]. Patients with HGPS exhibit a pronounced premature aging phenotype due to progerin accumulation [86]. The skin shows early aging signs, including thinning, decreased elasticity, and hyperpigmentation. Additionally, joint stiffness, atherosclerosis, and osteoporosis are common symptoms [86, 92, 94, 103]. The abnormal expression of progerin in these tissues leads to significant damage to nuclear structures and impairs tissue function [104]. Studies have demonstrated that progerin expression levels correlate with the severity of symptoms in HGPS patients [105]. High progerin expression exacerbates DNA damage, accelerates cellular senescence, and leads to more severe tissue degeneration [99]. Interestingly, progerin expression is also observed in normal aging processes, not just HGPS patients [106]. As age increases, low progerin levels gradually accumulate, suggesting that progerin may be part of the normal aging. Studies have shown that in normally aging human cells, progerin expression is associated with increased DNA damage and markers of cellular senescence [102]. This discovery has made progerin a critical target for research on normal aging. While abnormal progerin expression in HGPS triggers rapid aging, its gradual accumulation in normal cells may result in similar aging effects over time. This offers new insights into the molecular mechanisms of aging.

## Nesprin

Nesprins are a family of large transmembrane proteins primarily localized in the nuclear envelope. These proteins connect the cytoskeleton to the nucleoskeleton to form the LINC complex [107, 108]. Nesprins interact with KASH (Klarsicht, ANC-1, and Syne Homology) proteins, enabling the transmission of mechanical signals from the cytoskeleton to the nucleus, thereby regulating cellular mechanical stability and nuclear positioning [109]. The nesprin family includes four main members: nesprin-1, nesprin-2, nesprin-3, and nesprin-4. Although each nesprin has distinct structural and functional characteristics, they share a common KASH domain at the C-terminus, facilitating their insertion into the outer nuclear membrane and interaction with lamin proteins in the nucleoskeleton [110]. Additionally, these proteins typically possess an actin-binding domain at the N-terminus, which connects to the actin cytoskeleton, playing a role in nuclear positioning and mechanical signal transduction [111].

Nesprin-1 is the largest and most structurally complex member of the Nesprin family, with a full-length molecular weight of up to 1000 kDa [108]. Nesprin-1 contains multiple actin-binding domains, spectrin repeat sequences, and a C-terminal KASH domain [109]. The N-terminal actin-binding domain enables nesprin-1 to interact with actin filaments in the cytoskeleton, transmitting external mechanical stress to the nuclear membrane [112]. Nesprin-1 is localized in the outer nuclear membrane and functions within the space between the outer and inner nuclear membranes [110]. Through its interaction with Sad1-UNC-84(SUN) proteins in the LINC complex, nesprin-1 links the nucleoskeleton to the cytoskeleton, transmitting mechanical forces from the extracellular matrix and cytoskeleton to the nuclear membrane [113]. This mechanical signaling is critical for various cellular functions, including polarity, migration, nuclear positioning, and DNA repair [114]. Research indicates that nesprin-1 is key in maintaining structural stability and cell morphology [108]. Mutations or loss of nesprin-1 function can lead to cellular structural abnormalities, particularly in muscle and nervous system cells, which depend on precise mechanical transmission and nuclear positioning [115].

Nesprin-2, although similar to nesprin-1 in structure, differs in molecular size and tissue distribution. Nesprin-2 has a molecular weight of up to 800 kDa and shares the same N-terminal actin-binding domain and C-terminal KASH domain [116]. Nesprin-2 is expressed in various cell types, with particularly high levels in epithelial and fibroblast cells [117]. It is localized in the outer nuclear membrane and is closely associated with the NPC, which enables nesprin-2 to not only regulate nuclear positioning but also participate in nucleocytoplasmic transport [118]. By connecting to actin filaments, nesprin-2 helps transmit mechanical stress to the nucleus, influencing nuclear membrane stability and gene expression [108]. Furthermore, mutations in nesprin-2 are associated with structural abnormalities and genomic instability, particularly affecting cellular processes involving nucleocytoplasmic transport and nucleoskeleton stability [117]. Defects in nesprin-2 can lead to changes in nuclear morphology and are linked to degenerative diseases, including neurodegenerative disorders [114].

Nesprin-3 is a relatively simple but functionally distinct member of the Nesprin family [119, 120]. It is critical in linking the nuclear skeleton to the intermediate filament cytoskeleton via its interaction with intermediate filament-associated proteins, such as plectin [119, 121, 122]. A defining feature of nesprin-3 is its characteristic KASH domain, which anchors it to the outer nuclear membrane, where it interacts with lamins within the nuclear skeleton [123, 124]. Additionally, nesprin-3 binds to intermediate filament linker proteins like plectin through specific binding domains, facilitating the transfer of external mechanical signals to the nucleus [125, 126]. This process is crucial for regulating nuclear positioning and maintaining nuclear envelope morphology [107]. Under mechanical stress or external stimuli, the dynamic regulation of nesprin-3 influences cytoskeletal remodeling and the adaptability of the nuclear skeleton [127]. However, the expression of nesprin-3 decreases with age. This decline can result in nuclear envelope instability and a breakdown of the intermediate filament network, reducing DNA damage repair capacity and accelerating cellular aging. A loss of nesprin-3 function further suppresses mechanical signal transmission, leading to impaired cell migration and tissue regeneration.

Nesprin-4, the smallest member of the nesprin family, is uniquely associated with the microtubule system, distinguishing it from other nesprins [108, 128]. Its primary role is to regulate nuclear positioning and dynamics [129]. Like other nesprins, nesprin-4 contains a KASH domain, anchoring it to the outer nuclear membrane [120]. Its N-terminus interacts specifically with the dynein complex via unique binding domains, establishing a direct link to the microtubule cytoskeleton [130]. This interaction facilitates the dynamic regulation of nuclear spatial positioning, which is particularly crucial in dividing and migrating cells. Nesprin-4 is highly expressed in certain cell types, such as sensory epithelial cells, where it is critical for sensory function and mechanical adaptability. Defects in nesprin-4 lead to the mislocalization of nuclei and cell death in outer hair cells (OHCs), causing deafness in both humans and mice [112]. Additionally, as individuals age, nesprin-4 expression progressively decreases. This decline leads to abnormal nuclear positioning and disruption of mechanical signal transduction, especially in sensory neurons and epithelial cells, accelerating sensory degeneration, including hearing and vision loss [131].

The core function of the nesprin family is to link the cytoskeleton to the nucleus through the LINC complex, transmitting mechanical forces and regulating nuclear positioning [108]. This function is essential for maintaining cellular mechanical homeostasis, nuclear structural stability, and genomic regulation [109]. In various cell types, the nesprin family plays a significant role in maintaining nuclear morphology and regulating cell polarity [112]. Nesprin-1 and nesprin-2 are highly expressed in muscle cells, cardiomyocytes, and neurons, which rely on precise mechanical signal transmission to maintain normal function [110]. Dysfunction in nesprin is closely associated with the development of numerous diseases, including muscular dystrophy, cardiovascular diseases, and neurodegenerative disorders [115]. Recent research underscores the critical role of nesprin in maintaining nuclear envelope stability, facilitating mechanical signal transduction, and anchoring the cytoskeleton [108, 132, 133]. A decline in nesprin function has been identified as a major contributor to cellular and tissue degeneration associated with aging [134–136]. Further exploration of nesprins is essential, as it deepens our understanding of the molecular mechanisms underlying aging and provides a robust foundation for developing anti-aging therapies and treatments for age-related diseases [111, 137, 138].

## Emerin

The emerin gene (*EMD*) is located on Xq28 and consists of six exons and five introns [139, 140]. It encodes a protein of 254 amino acids, which includes an N-terminal nucleoplasmic domain of 220 amino acids, a C-terminal transmembrane domain of 23 amino acids, and an 11-residue luminal domain [141]. The N-terminal domain, located on the nucleoplasmic side of the nucleus, interacts with lamins, chromatin, and other proteins, forming the region where emerin carries out its principal functions [142]. These functions include maintaining nuclear morphology, regulating gene expression, and connecting to the cytoskeleton [38]. The C-terminal transmembrane region, a short hydrophobic sequence, anchors emerin to the inner nuclear membrane, ensuring its proper localization [143]. This region is critical for securing the N-terminal domain to the nuclear membrane.

Following synthesis, emerin is inserted into the endoplasmic reticulum (ER) during translation and subsequently diffuses through the ER to the nuclear membrane [144, 145]. Due to its small size (29 kD), emerin can freely diffuse through the NPC while remaining membrane-anchored [146]. Upon reaching the nucleus, emerin binds to A-type lamins [147]. In cells lacking A-type lamins, emerin shows increased mobility within the nuclear membrane and ER, suggesting that lamin binding is crucial for emerin’s stable localization [148, 149]. Emerin interacts with numerous binding partners, including transcriptional regulators and components of the LINC complex, enabling it to perform a variety of cellular functions [143]. These include gene expression regulation, cell signaling, and transduction of mechanical stress signals. Mutations in the emerin gene are associated with X-linked Emery-Dreifuss muscular dystrophy (X-EDMD) [150–153]. Approximately 95% of these mutations lead to the loss of emerin, which results in early contractures, progressively worsening muscle weakness, and cardiac conduction defects [152, 154].

In addition to the X-linked recessive form caused by *EMD*, autosomal dominant (involving *LMNA*) and autosomal recessive forms (involving *LMNA* and other rare genes such as *FHL1*, *TMEM43*, *SUN1*, *SUN2*, and *TTN*) have also been identified [153, 155]. Impaired muscle regeneration is a key contributor to the skeletal muscle defects observed in EDMD [156, 157]. For example, muscles from EDMD patients and emerin-deficient mice show increased expression of components involved in the muscle regeneration pathways, suggesting that emerin plays a critical role in suppressing these genes [158, 159]. Furthermore, the downregulation of emerin impairs myogenic differentiation in myoblasts [155, 160]. Recent studies have demonstrated that critical signaling pathways required for myogenic differentiation and skeletal muscle regeneration, including the Wnt, IGF-1, TGF-β, and Notch pathways, are disrupted in emerin-deficient myogenic progenitor cells [159, 161]. Additionally, emerin deficiency affects gene expression in the JNK, MAPK, NF-κB, and integrin signaling pathways [155, 158]. However, the mechanisms through which these disruptions lead to EDMD remain poorly understood [159, 160]. We propose that the underlying mechanisms may involve the following aspects: 1. Loss of mechanical stability: Deficiency or mutation of genes such as *EMD* and *LMNA* compromises the mechanical stability and integrity of the nuclear envelope [136, 162, 163]. This reduces the envelope’s ability to withstand external mechanical stress, resulting in progressive damage to muscle and cardiac cells during repeated contractions, ultimately causing functional loss [164]. 2. Impaired mechanical signal transmission: Dysfunction in the LINC complex, where emerin and lamin A/C play stabilizing roles, is a primary cause. Abnormalities in these components disrupt the mechanical coupling between the nuclear envelope and the cytoskeleton [158, 165]. Consequently, during muscle contraction or cardiac activity, mechanical forces are not effectively transmitted to the nucleus, leading to improper nuclear positioning [166]. This is particularly detrimental in multinucleated cells such as skeletal muscle fibers. 3. Alterations in nuclear morphology and chromatin structure: BAF, a small DNA-binding protein, interacts with double-stranded DNA, chromatin, and nuclear envelope proteins like emerin [156]. By binding to specific domains of emerin, BAF anchors chromatin near the nuclear envelope, stabilizing DNA and chromatin at the nuclear membrane. This interaction is essential for maintaining nuclear envelope repair and ensuring dynamic connectivity between the nuclear envelope and chromatin.

Emerin, along with Lap2B and MAN1, is a founding member of the LEM domain proteins, named for the LEM domain [167]. It binds to various transcriptional regulators, including GCL, Btf, Lmo7, β-catenin, SIKE, and BAF, modulating the expression of their target genes [149, 168, 169]. The absence of emerin disrupts gene expression in the JNK, MAPK, NF-κB, and integrin signaling pathways in both humans and mice [159]. Emerin also binds lamin A/C, forming an internal support structure that preserves nuclear morphology and protects nuclear integrity under mechanical stress [170]. In addition to its structural role, emerin regulates gene expression by binding to chromatin and influencing chromatin remodeling, thereby affecting specific gene activities [171, 172]. This regulatory function is particularly important in cardiomyocytes and skeletal muscle cells. Research shows that emerin can interact with transcription factors such as GCL and β-catenin, modulating gene activation or repression [149]. This indicates that emerin serves as both a structural and dynamic regulatory protein within gene networks [168, 173]. Furthermore, emerin regulates cytoskeletal organization and signaling pathways, interacting with protein kinases and other cytoskeleton-associated proteins, which are crucial for normal cell proliferation and differentiation [43, 174].

Studies suggest that emerin expression levels may decline significantly in aging cells [175]. This reduction could be part of the cell’s self-regulation mechanism or a consequence of age-related gene regulation [176]. In such cells, emerin may not anchor properly to the nuclear membrane, leading to functional impairments within the nucleus [177]. Mislocalization could further disrupt the nuclear lamina structure, gene expression, and nuclear morphology stability. Given emerin’s involvement in cellular aging, its dysfunction or mutations are linked not only to muscular dystrophies but also to other age-related diseases, such as cardiac and skeletal muscle degeneration [159, 169, 174, 178]. Abnormal expression or impaired function of emerin during cellular aging may accelerate the progression of these conditions. Emerin plays multiple essential roles in cellular aging, including maintaining nuclear structural integrity, regulating gene expression, and participating in cell cycle control and DNA repair processes [173, 179]. Abnormal emerin function or changes in its expression levels could serve as a significant marker and mechanism in cellular aging.

## SUN

SUN proteins were first identified in a conserved region of ~150 amino acids shared between Sad1 of *Schizosaccharomyces pombe* and UNC-84 of *Caenorhabditis elegans* [180–184]. In higher eukaryotes, these proteins contribute to the structural integrity of the NE by interacting with lamins and participating in the assembly of the LINC complex [185]. Notably, during the development of the mouse nervous system, SUN proteins interact with KASH proteins to regulate neuronal nuclear anchoring, a process essential for neuronal migration [183]. Moreover, SUN proteins are involved in regulating key signaling pathways, including MAPK and β-catenin, and play critical roles in various physiological processes, such as centrosome anchoring, telomere positioning, and spermatogenesis [127, 186, 187].

Classical SUN proteins are characterized by a conserved structural organization: their N-terminus is located within the nucleus, while their C-terminus is positioned in the nuclear membrane or the lumen of the endoplasmic reticulum, with the SUN domain specifically localized at the C-terminus [188, 189]. In addition to the SUN domain, classical SUN proteins typically contain coiled-coil domains, which facilitate their oligomeric assembly [190, 191]. This structural arrangement underpins their functional roles within the cell. In contrast, non-classical SUN family proteins, such as SUCO, SLP1, and SUN4, differ from classical SUN proteins by positioning their SUN domain between the N-terminus and C-terminus, rather than solely at the C-terminus [192–194]. This unique configuration suggests that non-classical SUN proteins may facilitate the formation of protein bridges that help maintain nuclear spacing. However, whether these proteins can bind KASH proteins or contribute to nuclear membrane fusion and the formation of intranuclear protein bridges remains an open question [183, 189, 190, 195, 196].

### Nuclear Localization

Experiments have shown that SUN1 and SUN2 are essential for properly localizing *Syne-1* and *Syne-2* to the nuclear membrane [162, 183, 195]. In mouse models with SUN1 and SUN2 knockout, the nuclear membrane localization of *Syne-1* and *Syne-2* is disrupted, resulting in defects in nuclear anchoring [197, 198]. Further studies have demonstrated that SUN1 and SUN2 interact with lamin A/C, forming a structural bridge that connects the nuclear lamina to the actin cytoskeleton, thus ensuring accurate nuclear positioning [59, 110]. This process is facilitated by the LINC complex, which links lamin A in the inner nuclear membrane to the cytoskeleton in the outer nuclear membrane, maintaining the integrity of the nuclear envelope [109, 129]. Transmission electron microscopy has revealed that the loss of *SUN1* and *SUN2* causes abnormal swelling of the outer nuclear membrane, likely due to the LINC complex’s crucial role in regulating nuclear membrane morphology and the spacing between the inner and outer nuclear membranes [199]. Additionally, SUN3-5 contain fewer coiled-coil residues [80–130] compared to SUN1 and SUN2 (~300), leading to narrower spacing between the two nuclear membranes in spermatogenic cells [129, 200]. SUN3 is specifically localized to the nuclear membrane of spermatogenic cells, where it plays a role in nuclear membrane remodeling through interactions with microtubules [201, 202]. Furthermore, SUN4 is critical for directing the movement of the centrosome toward the nuclear membrane, underscoring its importance in cellular architecture during spermatogenesis [203, 204].

### Neural Development and Cell Migration

In mice, the interaction between *SUN1/2* and *Syne-1/2* is crucial for proper neuronal development and migration [198]. The loss of *SUN1/2* or *Syne-1/2* disrupts neuronal radial migration, resulting in impaired nuclear translocation and significant neurodevelopmental abnormalities [205, 206]. Furthermore, knockout models exhibit retinal defects, including thinning of the outer nuclear membrane, mislocalization of photoreceptor nuclei, and impaired retinal electrophysiological responses, highlighting the pivotal role of these proteins in maintaining retinal structure and function [207–210].

### DNA Damage Repair and Signaling Pathways

SUN proteins not only interact with KASH proteins but also stabilize the nuclear structure through interactions with lamin A/C [211]. Mutations in lamin A/C are known to cause defects in DNA damage repair, compromising genome stability [212]. In HGPS, mutations in lamin A/C weaken its interaction with SUN1/2, suggesting that SUN1/2 may play a critical role in maintaining genome stability [213]. Studies have shown that SUN1/2 can bind to DNA damage repair-related molecules, including DNA-PKcs, Ku70, Ku80, and Rcn2, thereby regulating the MAPK signaling pathway [214]. Upon DNA damage, DNA-PKcs and Ku70 anchor DNA to the nuclear membrane, and the SUN1/2-Rcn2 complex activates ERK1/2 [215]. Once phosphorylated, ERK1/2 enters the nucleus and further phosphorylates DNA damage signaling proteins, such as ATM, ATR, and γ-H2AX, enabling rapid DDR [216]. By constructing stable SUN1-expressing cell lines and performing mass spectrometry analysis, researchers identified several proteins interacting with SUN1, predominantly localized to the nuclear membrane and involved in DNA repair. The double knockout of *SUN1* and *SUN2* in mouse cells led to a significant delay in ERK1/2 phosphorylation, indicating that *SUN1* and *SUN2* activate the mitogen-activated protein kinase (MAPK) signaling pathway via Rcn2, playing a critical role in DDR [217].

## LINC: Interaction of Nesprin with Lamin

The interaction between nesprin and lamin through the LINC complex is critical for mechanical force transmission and maintaining nuclear skeleton stability in cells [218]. Serving as a molecular bridge across the nuclear membrane, the LINC complex connects the cytoskeleton outside the nucleus to the nuclear skeleton within [109]. This interaction is essential not only for preserving cellular structure and responding to external mechanical stress but also for modulating gene expression, cell signaling, and DNA repair [39, 56, 61, 109, 110]. The LINC complex consists of two main components: nesprins in the outer nuclear membrane and SUN proteins in the inner nuclear membrane [195]. Nesprin binds to the N-terminus of SUN proteins via its C-terminal KASH domain, allowing it to span the nuclear membrane and establish a connection between the cytoskeleton and the nuclear skeleton [183, 219]. Inside the nucleus, lamin A/C forms the core of the nuclear skeleton, organizing a tightly-knit network that stabilizes nuclear morphology and supports various nuclear functions [39, 59, 63]. By forming this “mechanical bridge,” the LINC complex facilitates the interaction between nesprins and lamins, ensuring the transmission of external mechanical forces to the nuclear skeleton [39, 56, 63, 65, 218]. This mechanical signal transmission enables the regulation of nuclear shape and internal functions in response to mechanical cues from the cellular environment [59, 108, 110, 220]. Cells are constantly exposed to external mechanical forces, including those generated by extracellular matrix pressure, fluid shear stress, and tensile forces. These forces are transmitted through the cytoskeleton to the LINC complex, which relays them to lamin within the nuclear skeleton [221]. The LINC complex thus enables cells to detect and respond to mechanical changes in their environment, influencing key cellular processes such as migration, differentiation, and proliferation [108–112]. When a cell is subjected to external forces, the LINC complex enables the nucleus to adapt its shape in response to varying mechanical conditions [185]. This adaptation is facilitated by the reorganization of the lamin meshwork, which plays a key role in preserving nuclear stability during these changes. Mechanical forces transmitted via the LINC complex can alter the structure of the nuclear skeleton, leading to downstream effects on chromatin organization [222]. For example, external forces can influence the interaction between lamin and chromatin, altering the chromatin’s open or closed state, thereby regulating gene expression [223]. This mechanism is especially crucial during processes such as cell differentiation and tissue formation [224].

### Effect of LINC Complex Dysregulation on Nuclear Skeleton Stability

During aging, significant changes occur in the expression levels and structural integrity of nuclear scaffold proteins, including lamin A/C [39, 61, 63, 103, 218]. Mutations or downregulation of lamin A can impair LINC complex function, leading to decreased nuclear lamina stability and abnormalities in nuclear membrane morphology [225]. One hallmark of cellular aging is the alteration of nuclear morphology, such as wrinkling of the nuclear membrane and changes in nuclear pore complex density, often linked to dysfunction of lamin and the LINC complex [226].

The decline in nuclear scaffold stability not only alters nuclear membrane morphology but also disrupts the regulation of gene expression and chromatin organization within the nucleus. As lamin A/C functionality diminishes, its ability to anchor chromatin weakens, resulting in increased heterochromatinization and subsequent gene expression abnormalities [178]. These changes are closely associated with the activation of aging-related genes (e.g., p16 and p21), further promoting cellular senescence [227].

### Effects of LINC Complex Dysregulation on Mechanical Force Transduction

Dysregulation of the LINC complex impairs the cell’s ability to sense and respond to external mechanical forces [221]. In aging cells, reduced sensitivity to mechanical environments hinders their ability to adapt to varying mechanical conditions. This dysfunction likely arises from the impaired function of the LINC complex, which compromises the mechanical connection between the cytoskeleton and the nuclear skeleton, disrupting the transmission of mechanical signals to the nucleus [109, 185, 218]. Impaired mechanical force transmission not only alters nuclear morphology but also disrupts a range of cellular functions that rely on mechanical signaling, such as cell migration and proliferation. This impairment is particularly pronounced in aging tissues, including the skin, heart, and muscle, where cells show a significantly diminished capacity to respond to mechanical stress.

### Effect of LINC Complex Dysregulation on DNA Damage Repair

Dysregulation of the LINC complex compromises the cell’s ability to repair DNA damage.231 In aging cells, DNA damage accumulation is a hallmark of cellular aging, and lamin A/C is critical in the DNA repair process [178]. Research indicates that lamin A/C, beyond being a fundamental component of the nuclear scaffold, is involved in regulating DNA repair mechanisms such as non-homologous end joining (NHEJ) [228].

When the LINC complex is disrupted, the interaction between lamin and DNA repair factors may be compromised, reducing DNA repair efficiency. As DNA damage accumulates, cells progressively lose their capacity to proliferate and enter a state of irreversible senescence [29]. Moreover, the accumulation of DNA damage triggers the activation of tumor suppressor genes like p53, further accelerating the onset of senescence-related phenotypes [229].

## Mechanism Discussion

### Lamin and Cellular Senescence

Current research suggests that lamin plays a crucial role in cellular processes through at least three potential mechanisms. First, lamin regulates gene expression and cell differentiation by controlling the spatial positioning of genes within the nucleus and through epigenetic modulation. Second, lamin is essential for the repair of DNA damage and the maintenance of genomic stability. Lastly, lamin contributes to the modulation of transcription factors and signaling components that govern various differentiation pathways.

#### Gene Expression and Epigenetic Modulation

Dysregulation of epigenetic control pathways in progeroid cells results in reduced levels of facultative and specific chromatin markers, such as H3K27me3 and H3K9me3 histone modifications, along with an increase in H4K20 trimethylation [230–232]. These alterations are accompanied by significant changes in lamin-chromatin interactions, genome organization, and the distribution of H3K27me3 at the genomic level in progeroid cells [233]. However, not all studies have observed these changes consistently [232, 234, 235], underscoring the need for further investigation. Additionally, DNA methylation profiles in progeroid cells are altered, further indicating epigenetic instability [236].

In myopathic cells with lamin mutations, the spatial organization of chromosomes is significantly disrupted, potentially affecting gene expression [89, 237, 238]. Research in *Caenorhabditis elegans* has shown that during muscle differentiation, the entire genomic DNA, including muscle development promoters, relocates from the nuclear periphery to the nuclear center. However, the expression of lamin mutations associated with muscle disease impedes this relocation, thereby interfering with the internalization of muscle-specific genes and muscle differentiation [235, 239].

The molecular pathways through which lamin mutations influence epigenetic patterns and gene positioning remain unclear. One hypothesis suggests defects in the nucleosome remodeling and deacetylase (NuRD) complex, a critical multi-subunit protein complex involved in chromatin remodeling and gene regulation. The NuRD complex performs two essential functions: ATP-dependent chromatin remodeling and histone deacetylation, allowing it to modulate DNA accessibility for transcription factors and other regulatory proteins. This modulation ultimately determines whether a gene is silenced or activated. Studies indicate that defects in the NuRD complex contribute to chromatin abnormalities in progeroid cells [232, 240–243].

Furthermore, genomic stability relies on the efficient repair of DNA damage induced by both internal metabolic processes and external factors, such as radiation and drugs [33]. Additionally, lamin may contribute to genomic stability by supporting the structure and function of telomeres. The loss of lamin A/C [244–246]and the expression of progerin [247, 248]have been associated with altered nuclear positioning of telomeres and/or telomere shortening, further compromising genomic integrity.

#### Repair of DNA Damage and the Maintenance of Genomic Stability

Current research underscores the complex role of lamin A/C in the repair of double-strand DNA breaks (DSBs) [179]. DSB repair typically occurs through two relatively independent pathways: the error-prone NHEJ pathway and homologous recombination, the latter of which uses sister chromatid genetic information to repair DNA damage with high fidelity during the S and G2 phases of the cell cycle [33, 249]. Studies involving cells from progeria patients [35, 250] and progeroid mouse models lacking the prelamin A-processing enzyme Zmpst24 [251, 252] have revealed an increased incidence of DSBs [179].

Furthermore, DSBs induced by UV and γ-radiation exhibit deficiencies in the recruitment of essential DNA damage repair factors, such as 53BP1 [251], Rad50, and Rad51 [253], as well as the kinases ATM (ataxia-telangiectasia mutated) and ATR (ATM and Rad3-related), at damage sites [254]. In contrast, the Xeroderma pigmentosum group A (XPA) protein, a critical factor for nucleotide excision repair, is recruited normally to these sites [253]. Notably, smooth muscle cells derived from induced pluripotent stem cells of progeria patients display reduced levels of PARP-1 (poly ADP-ribose polymerase), a protein that inhibits the NHEJ pathway. This reduction leads to a greater reliance on the error-prone NHEJ pathway, resulting in the accumulation of chromosomal defects and increased cell death in mutant cells [255]. Additionally, fibroblasts in progeroid mice upregulate the p53 tumor suppressor pathway, driving cellular senescence and accelerating organismal aging. In these mice, inactivating the p53 gene partially rescues these aging phenotypes [179, 252].

Recent research further suggests that lamin A/C contributes to DNA stability by providing a scaffold at damage sites through its interaction with DSB-induced phosphorylated γ-H2AXl [256]. Lamin A/C may also help maintain stable protein levels of 53BP1, a critical component of the NHEJ pathway, by preventing the transcriptional upregulation of cathepsin L, a cysteine protease that targets 53BP1 [257, 258]. Functional lamin A/C appears essential for the NHEJ pathway [249]. Interestingly, MCF7 cells lacking lamin A/C show a 40% reduction in homologous recombination, likely due to the transcriptional repression of BRCA1 and RAD51 proteins [257]. However, it remains unclear whether this inhibition of BRCA1 and RAD51 is a direct or indirect effect of lamin A/C.

In addition to impairments in DSB repair within lamin A/C-mutant cells, progerin expression also promotes DNA damage accumulation by increasing reactive oxygen species [259]. Fibroblasts from HGPS patients and cells expressing prelamin A, derived from *ZMPSTE24*-deficient mice, exhibit elevated levels of spontaneous DNA damage, indicated by increased baseline levels of γ-H2AX [251, 252, 260]. *LMNA*-deficient cells also display spontaneous γ-H2AX foci, chromosomal or chromatid breaks, and increased aneuploidy, suggesting that lamin A may play an active role in DNA repair [246, 261].

#### Modulating Transcription Factors and Signaling Components

Numerous studies have highlighted the critical role of lamin in various signaling pathways, where it directly interacts with key signaling factors. Through these interactions, lamin can influence transcription by either recruiting transcriptional activators and repressors to distant promoters or by providing a structural scaffold that facilitates the effective interaction and activation of signaling molecules, thereby supporting signal transduction [262, 263]. Consequently, the integrity of many signaling pathways is compromised in cells lacking lamin or in those expressing lamin mutations associated with the disease.

The Rb protein regulates the cell cycle by inhibiting the E2F-DP3 transcription factor complex, which is essential for initiating gene expression required for S phase entry [264–267]. Studies suggest that the function of Rb as a transcriptional repressor depends on its association with the nuclear structure, and mutations in its COOH terminus disrupt its nuclear binding [268–271]. Rb is anchored to filamentous structures within the nuclear scaffold, which are similar in size to intermediate filaments [272]. Evidence for the involvement of A-type lamin in these nuclear structures includes two key findings: first, A-type lamin forms a salt-resistant complex with LAP2 in the nuclear scaffold [273];second, second, this complex binds to Rb via its C-domain during the G1 phase of the cell cycle, which appears to serve as a protective mechanism for Rb [274]. In fibroblasts from *LMNA -/-* mice, Rb becomes a target for proteasomal degradation, resulting in growth characteristics similar to those observed in *Rb -/-* fibroblasts [275].

Recent studies have identified a direct interaction between lamin A/C and c-Fos. This interaction inhibits AP-1 activity and leads to a reduction in the proliferation of mouse embryonic fibroblasts. Conversely, cells lacking A-type lamin exhibit increased proliferation rates [85]. Lamin A/C interacts with LAP2α [274], forming a protein complex that stabilizes the tumor suppressor protein Rb, a key regulator of cell proliferation [275, 276 Lamin A also provides a scaffold that facilitates protein phosphatase 2a-dependent dephosphorylation of Rb, a process essential for cell cycle arrest [277]. In contrast, ERK1/2, a component of the MAPK pathway, binds to A-type lamin and releases Rb from the lamin complex in a kinase-independent manner, enabling cyclin-dependent kinase-mediated phosphorylation and thereby promoting cell cycle progression [278]. The impairment of the Rb pathway has been observed in the muscle tissue of EDMD patients [279] and in progeroid cells [280]. Additionally, lamin A influences cell proliferation by regulating c-Fos, a direct downstream transcription factor of the MAPK pathway. In the absence of mitotic signals, c-Fos, a component of the AP-1 complex, is recruited to the nuclear lamina, which inhibits AP-1-dependent transcription [85]. Under mitogenic stimulation, phosphorylated ERK1/2 associates with the membrane, subsequently phosphorylating and activating c-Fos, which leads to a rapid primary c-Fos-dependent transcriptional response [281]. Interestingly, in mice expressing the myogenic lamin A mutant (H222P) and in patients with DCM, the ERK and p38α MAPK pathways are upregulated [282, 283]. In these models, reducing MAPK signaling or deleting ERK1 can restore normal cardiac phenotypes [284, 285]. However, it is still unclear whether this upregulation of the ERK pathway is a direct consequence of lamin A mutation or a secondary effect of stress-induced signaling.

Given that many signaling pathways affected in laminopathies regulate cell proliferation and differentiation, and that adult stem cells are impacted in several cell and mouse models [286–289], these findings support the hypothesis that an imbalance in adult stem cell proliferation and differentiation may lead to stem cell exhaustion or impaired tissue renewal, thereby contributing to specific tissue pathologies [290, 291]. In support of this hypothesis, progeroid cells have been found to exhibit excessive proliferation and premature senescence [292, 293]. A recent study suggests that lamin A interacts with the deacetylase SIRT1, and that reduced deacetylase activity in progeroid mice lacking Zmpste24 leads to adult stem cell depletion [294]. In progeria models derived from induced pluripotent stem cells, differentiation of mesenchymal stem cells and smooth muscle progenitor cells is impaired [295, 296]. This is consistent with previous findings of progressive smooth muscle defects in progeroid mouse model [297], and a recent report showing that prelamin A expression in vascular smooth muscle cells induces calcification through the activation of the senescence-associated secretory phenotype [298].

### Nesprin and Cellular Senescence

The role of nesprin in cellular aging is closely linked to the DDR, mechanotransduction, mitochondrial function, and various signaling pathways. Persistent DNA damage is a primary driver of cellular senescence [18, 34]. Research has shown that nesprin interacts with the DDR mechanism in multiple ways, including with mismatch repair proteins MSH2 and MSH6, as well as the NHEJ repair pathway [299]. In hepatocellular carcinoma cell lines, Hep3B and Huh7, nesprin expression is significantly reduced, accompanied by elevated baseline levels of DNA damage, as indicated by γ-H2AX staining, compared to normal liver cells [299]. Knocking out nesprin in normal liver cells leads to a substantial accumulation of γ-H2AX staining, indicating increased DNA damage. Conversely, ectopic expression of nesprin in Hep3B and Huh7 cells reduces DNA damage levels [177]. Similar outcomes are observed in the mouse embryonic mesenchymal stem cell line (CH310T1/2) and normal human dermal fibroblasts (NHDFs), where nesprin knockdown results in an increase in DNA double-strand breaks [299]. These results are consistent with studies in fission yeast (*S. pombe*), where the nesprin homolog Kms1 is essential for double-strand break repair [300]. Studies have also shown that in vascular smooth muscle cells (VSMCs), nesprin binds to ERK1/2, facilitating the accumulation of signaling molecules in specific nuclear regions to coordinate the DDR process [138, 301]. Nesprin specifically interacts with lamin A/C in the nuclear membrane and PML nuclear bodies, creating a distinct ERK1/2 partition near regions of DNA damage [302]. This partition not only promotes the localization of DDR components but also provides structural support essential for DDR signal transduction [32]. Remarkably, when prelamin A accumulates, the local partitioning of ERK1/2 intensifies, highlighting its role as a critical regulator of cellular aging and DNA repair [250]. Additionally, the removal or disruption of nesprin or lamin A/C affects ERK1/2 partitioning within PML bodies, misaligning ATM protein at DNA damage sites, and weakening Chk2 signaling [138, 303]. This impaired DDR response increases the vulnerability of VSMCs to genomic instability, accelerating cellular aging. Overall, by forming a complex with lamin and ERK1/2, nesprin maintains the spatial integrity and signaling precision of DDR during cellular aging, ensuring efficient DNA repair and controlled cell cycle progression, and playing a pivotal role in the aging process [304].

Nesprin is essential for cellular mechanotransduction, conveying mechanical signals from the extracellular environment to the nucleus and regulating cellular behavior [108–110, 113, 177, 299]. In senescent cells, significant changes occur in mechanical properties, such as increased cellular stiffness, and nesprin-mediated mechanotransduction becomes impaired, potentially diminishing cellular responses to mechanical stimuli [106, 185, 218, 305]. As a mechanosensor, nesprin transfers forces from the actin cytoskeleton to the nucleus, thereby influencing nuclear shape and chromatin dynamics [108, 110, 177, 306]. During aging, a decline in nesprin expression weakens cellular responsiveness to mechanical cues, affecting nuclear mechanical stability and leading to age-related changes in chromatin organization and gene expression profiles [138, 302]. This impairment in mechanotransduction also reduces the activation of mechanosensitive genes that are vital for maintaining cellular function and adapting to mechanical stress [138, 250]. Additionally, age-associated changes in cellular stiffness exacerbate nesprin’s functional decline, intensifying nuclear deformation and accelerating cellular senescence. In senescent cells, mechanotransduction becomes abnormal, partly due to disruptions in the LINC complex, primarily caused by nesprin dysfunction [307, 308]. This disruption hampers the effective transmission of forces from the extracellular matrix (ECM) to the nucleus, resulting in impaired nuclear signaling and reduced cellular homeostasis [37, 309]. Moreover, impaired nesprin-mediated mechanotransduction is closely linked to diminished YAP/TAZ signaling activation, which is crucial for cell proliferation and survival, thus further accelerating cellular senescence [218, 310, 311].

Recent studies have highlighted the critical role of nesprin in regulating mitochondrial function, which is essential for cellular energy metabolism [312, 313]. Nesprin dysfunction is closely associated with alterations in mitochondrial dynamics, specifically reduced fusion and increased fragmentation, phenomena commonly observed in senescent cells. Nesprin regulates mitochondrial morphology and distribution through interactions with Bcl-2 family proteins and caspases. During apoptosis, decreased nesprin-2 expression disrupts the regulation of Bcl-2 family proteins, leading to impaired inhibition of pro-apoptotic factors such as Bax and Bak. This dysregulation facilitates mitochondrial outer membrane permeabilization (MOMP), a key event in the intrinsic apoptotic pathway. MOMP contributes to mitochondrial fragmentation and loss of membrane potential, thereby reducing ATP production capacity [312, 314]. These mitochondrial alterations further exacerbate energy deficits and increase ROS generation, which in turn accelerates cellular aging and functional decline.

Additionally, nesprin is involved in signaling pathways that regulate cellular senescence [144, 309]. For instance, nesprin plays a role in regulating the PI3K/AKT pathway, which is essential for cell survival and proliferation. Nesprin dysfunction disrupts this pathway, accelerating cellular senescence and leading to the accumulation of senescent cells in aging tissues [315]. Specifically, decreased nesprin levels impair AKT activation, reducing the phosphorylation of downstream targets that are critical for cell cycle progression and survival. Consequently, more cells enter a senescent state characterized by irreversible growth arrest and the secretion of pro-inflammatory cytokines, ultimately resulting in tissue dysfunction [16, 315].

## Conclusion

This study provides a comprehensive analysis of the critical roles of nuclear envelope proteins, particularly nesprins and lamins, in the mechanisms underlying cellular senescence. As integral components of the nuclear envelope, nesprins and lamins play pivotal roles in maintaining nuclear structure, mediating mechanical signal transduction, and regulating chromatin organization and gene expression. Dysfunctions in these proteins, as highlighted in this review, are closely linked to the acceleration of aging-related phenotypes and the development of age-associated pathologies, including HGPS and various degenerative diseases. The synergistic interactions between lamins and nesprins through the LINC complex emphasize their combined contributions to nuclear stability, DDR, and mechanotransduction. Additionally, research on progeria syndromes has provided significant insights into the molecular mechanisms driving both pathological and physiological aging. Finally, our findings underscore the importance of further investigation into the regulatory pathways associated with nuclear envelope proteins, as these efforts may uncover new therapeutic targets for combating cellular senescence and treating age-related diseases.
